# *Lactobacillus murinus* Reduces Susceptibility to Secondary MRSA Infection in IAV-Infected Mice Through Promoting a T Cell-Independent IgA Response

**DOI:** 10.3390/microorganisms13071709

**Published:** 2025-07-21

**Authors:** Qichao Chen, Yanfeng Lin, Kaiying Wang, Jinhui Li, Peng Li, Hongbin Song

**Affiliations:** 1 Department of Pulmonary and Critical Care Medicine, The Affiliated Hospital of Yangzhou University, Yangzhou University, Yangzhou 225000, China; cqc0606yz@163.com; 2 Chinese PLA Center for Disease Control and Prevention, Beijing 100071, China; llyf8180@163.com (Y.L.); wkylibra@163.com (K.W.); jinhuili311@163.com (J.L.)

**Keywords:** secondary methicillin-resistant *Staphylococcus aureus* infection following influenza A virus, *Lactobacillus murinus*, gut microbiota, T cell-independent IgA response

## Abstract

Secondary methicillin-resistant *Staphylococcus aureus* (MRSA) infection causes high mortality in patients with influenza A virus (IAV). Our previous study observed that the relative abundance of *Lactobacillus murinus* (*L. murinus*) was significantly reduced in both the respiratory tract and gut of IAV-infected mice and negatively correlated with the severity of IAV–MRSA coinfection pneumonia, but the role of *L. murinus* remains unclear. Here, we supplemented the respiratory tract and gut of IAV-infected mice with live *L. murinus* and performed a secondary MRSA infection challenge to investigate the effects and potential mechanisms further. Data showed that *L. murinus* supplementation significantly reduced mortality and pathogen loads in IAV–MRSA coinfected mice and upregulated the lung T cell-independent (TI) IgA response in IAV-infected mice. The 16S rRNA gene sequencing results showed that *L. murinus* supplementation ameliorated microbiota composition disorder and regulated metabolic dysfunction in the gut of IAV-infected mice. The correlation analysis and antibiotic cocktail treatment experiment showed that the TI IgA response in lungs is dependent on gut microbiota. These findings demonstrated that *L. murinus* supplementation reduces susceptibility to secondary MRSA infection in IAV-infected mice by promoting the TI IgA response, and provide a new perspective on the use of probiotics to prevent secondary bacterial infection following IAV infection.

## 1. Introduction

The influenza pandemic is a serious threat to global public health [1]. According to the World Health Organization, seasonal influenza epidemics result in 3–5 million cases of severe illness and up to 650,000 respiratory deaths annually [2]. Studies have reported that up to 30% of severe influenza cases requiring ICU care involve bacterial coinfections, with *Staphylococcus aureus* being the most frequently isolated organism [3]. Secondary methicillin-resistant *Staphylococcus aureus* (MRSA) infection following influenza A virus (IAV) infection leads to high mortality in IAV-infected patients [4]. The normal microbiome protects against respiratory tract infections by resisting pathogen colonization and regulating immune response; however, host factors and external interventions can induce dysbiosis, thereby increasing susceptibility to pneumonia [5]. In our previous studies, the relative abundance of *Lactobacillus murinus* (*L. murinus*) was significantly reduced in both the respiratory tract and gut of IAV-infected mice, and this reduction correlated negatively with the severity of IAV–MRSA coinfection [6,7]. As a potential probiotic, *L. murinus* has been reported to have anti-inflammatory and immunomodulatory functions. For example, the supplementation of *L. murinus* ASF361 has been demonstrated to inhibit the outgrowth of *S. pneumoniae* in IAV-infected mice [8]. *L. murinus* CNCM I-5314 supplementation increased Th17 and RORγt+ regulatory T cells levels in lung tissue and reduced lung inflammation in tuberculosis patients [9]. Moreover, *L. murinus* has proven effective in alleviating intestinal ischemia/reperfusion injury through promoting the release of interleukin-10 from M2 macrophages [10]. However, the role and mechanisms by which *L. murinus* modulates susceptibility to secondary MRSA pneumonia following IAV infection remain unclear.

Secretory IgA (SIgA) is a major immunoglobulin present on mucosal surfaces and resists pathogen invasion by preventing the attachment of foreign microorganisms, promoting colonization by commensal microorganisms, and influencing bacterial gene expression and function [11]. SIgA is composed of IgA monomers produced primarily by the T cell-dependent (TD) and T cell-independent (TI) IgA pathways. In the presence of interleukin-21 (IL-21), the CD40 ligand (CD40L) of follicular helper T cells engages with CD40 on antigen-specific B cells, inducing antibody class-switching recombination of plasma cells to produce highly specific IgA. The TI IgA pathway involves microbial-induced innate CD40L-like molecules known as the TNF family B-cell activating factor (BAFF) and A proliferation-inducing ligand (APRIL). The BAFF/APRIL produces systemic IgA through the TI IgA pathway by binding to the transmembrane activator and the CAML interactor (TACI) on B cells. IgA produced by the TI IgA pathway is polyreactive and binds to a wide range of bacteria [12]. Many studies have shown that *Lactobacillus* spp. play an important role in local and systemic IgA secretion and type switching. For example, *Lactobacillus rhamnosus* supplementation significantly increased stool SIgA levels in healthy volunteers [13], and the oral intake of *Lactobacillus casei* Shirota decreased serum pro-inflammatory cytokine levels and increased salivary SIgA in humans [14]. Interestingly, our previous studies found that the relative abundance of *L. murinus* was positively correlated with lung B cells level and significantly negatively correlated with spleen B cells level in IAV–MRSA coinfection mice [6]. We therefore hypothesized that *L. murinus* may regulate susceptibility to secondary MRSA infection following IAV infection by modulating the host IgA response.

Here, we supplemented the respiratory tract and gut of IAV-infected mice with live *L. murinus* and performed a secondary MRSA infection challenge. We investigated the effects of *L. murinus* supplementation on susceptibility to secondary MRSA infection, lung IgA response, gut and lower respiratory tract microbiota in IAV-infected mice, and elucidated the relationship between lung IgA response and microbiota. Further, we used an antibiotic cocktail treatment experiment to clarify the relationship between gut microbiota and lung IgA response. Our study will provide a new probiotic therapy for the prevention of secondary bacterial infection following IAV infection.

## 2. Materials and Methods

### 2.1. Virus and Bacteria

The influenza virus strain A/Puerto Rico/8/34 (H1N1) (PR8) was amplified using embryonated chicken eggs and quantified using a 50% tissue culture infective dose (TCID50) assay in Madin–Darby canine kidney cells as described previously [15]. MRSA strain BJFAN203 was cultured in standard LB broth (Solarbio, Beijing, China) for 5 h. The *L. murinus* strain CICC 23140 was grown in MRS broth (Solarbio, Beijing, China) for 12 h. Colony-forming units (CFU) of MRSA or *L. murinus* strains were quantified using the standard plate counting method in LB or MRS plates (Solarbio, Beijing, China) as described previously [16]. The influenza virus strain A/Puerto Rico/8/34 and MRSA strain BJFAN203 were maintained in our laboratory. The influenza virus strain A/Puerto Rico/8/34 was identified by RT-PCR and Sanger sequencing. The MRSA strain BJFAN203 was confirmed by high-throughput sequencing (GenBank accession number JAPKAA000000000). *L. murinus* CICC 23140 was purchased from the China Center of Industrial Culture Collection, Beijing, China) and originally isolated from the feces of healthy mice. The virus and bacteria were pelleted down, washed, and diluted with PBS prior to animal experiments.

### 2.2. Animal Experiments

Specific pathogen-free (SPF) C57BL/6N mice (female, 6–8 weeks) were purchased from Beijing Vital River Laboratory Animal Technology Co., Ltd. (Beijing, China) and housed under SPF/BSL2 conditions. All animals were kept under a strict 12 h light/dark cycle for 7 days to adjust to housing conditions before experiments. The methodology for establishing the murine model of secondary MRSA infection following IAV infection is shown in Figure S1. For the *L. murinus* supplementation experiment, mice were randomly divided into three groups (25 mice per group): (1) Mock group; (2) IAV–MRSA coinfection group; (3) IAV–MRSA coinfection + *L. murinus* group. The Mock group of mice were infected with 25 µL of PBS on days 0, 2, and 4 by nasal drip. The IAV–MRSA coinfection group of mice were infected with 25 µL of PR8 (10^1.6^ TCID50/mL), PBS and MRSA (6 × 10^9^ CFU/mL) on days 0, 2, and 4 by nasal drip. The IAV–MRSA coinfection + *L. murinus* group were given *L. murinus*-supplemented water (1 × 10^9^ CFU/mL) from day 0 to day 4 as previously reported, and were inoculated with 25 µL of PR8 (10^1.6^ TCID50/mL), *L. murinus* (2 × 10^9^ CFU/mL) and MRSA (6 × 10^9^ CFU/mL) on days 0, 2, and 4 by nasal drip. Animals were deeply anesthetized with 0.3% pentobarbital sodium (intraperitoneal injection, 50 mg/kg) during the infection experiment (Figure S1B). Coinfected mice (*n* = 10) were used to calculate survival rates at 7 days post-infection. For the antibiotic cocktail treatment experiment, mice were randomly divided into two groups (10 mice per group): (1) Control (CON) group; (2) Antibiotic cocktail treatment group (ABX). The control group of mice were given normal water. The ABX group of mice were given ABX water (ampicillin 1 mg/mL; neomycin 1 mg/mL; metronidazole 1 mg/mL; and vancomycin 0.5 mg/mL) for 2 weeks to deplete the gut microbiota as previously reported [17]. Drinking water containing *L. murinus* or antibiotics was replaced every 24 h to maintain bacterial viability and ensure consistent treatment. All mice had ad libitum access to water and food throughout the experiment period. The animals were housed under controlled environmental conditions, with the temperature maintained at 22 ± 2 °C and a strict 12 h light/dark cycle. Upon reaching the experimental stage, animals were euthanized using 0.3% pentobarbital sodium (intraperitoneal injection, 200 mg/kg). Bronchoalveolar lavage fluid (BALF) was collected by washing the bronchoalveolar tree three times using 1 mL sterile PBS. Fecal, serum, and lung samples were collected in a sterile manner for further examination.

### 2.3. Viral and Bacterial Load Detection

Total RNA of BALF samples were extracted using a QIAamp viral RNA mini kit (Qiagen, Hilden, Germany) according to the manufacturer’s instructions. The IAV load of BALF samples were quantified using Luna universal probe one-step reverse transcription-qPCR kit (NEB, Ipswich, MA, USA) according to the manufacturer’s instructions. The cycling conditions were: 55 °C for 10 min; 95 °C for 1 min; 40 cycles of 95 °C for 10 s, 60 °C for 30 The following primers of IAV were: 5′-GACCRATCCTGTCACCTCTGAC-3′ (Forward primer), 5′-GGGCATTYTGGACAAAKCGTCTACG-3′ (Reverse primer) and 5′-FAM-TGCAGTCCTCGCTCACTGGGCACG-BHQ1-3′ (Probe, Melbourne, VIC, Australia). Copies of IAV were determined using a standard curve. The MRSA loads in BALF samples were quantified by the standard plate counting method and reported as CFU per milliliter of BALF. Samples were cultured on *Staphylococcus aureus* chromogenic medium (HOPEBIO, Qingdao, China) and incubated at 37 °C for 12 h on 90 mm plates. For the antibiotic cocktail treatment experiment, total DNA of fecal samples was extracted using a QIAamp PowerFecal Pro DNA Kit (Qiagen, Hilden, Germany) according to the manufacturer’s instructions. Total bacterial load of fecal and BALF samples was quantified using Luna Universal Probe qPCR Master Mix (NEB, Ipswich, MA, USA) according to the manufacturer’s instructions. The following primers of 16S rDNA were recommended as previously described [18]: universal forward primers 8FM (5′-AGAGTTTGATCMTGGCTCAG-3′) and 8FB (5′-AGGGTTCGATTCTGGCTCAG-3′), reverse primer Bact515R (5′-TTACCGCGGCKGCTGGCAC-3′) and TaqMan probe Bact338K (5′-FAM/CCAKACTCCTACGGGAGGCAGCAG/TAMRA-3′). Relative quantification was calculated by division with the control group.

### 2.4. Hematoxylin and Eosin Staining

Fresh right lung tissues were collected under sterile conditions and fixed in paraformaldehyde 4% solution for 1 day. The prepared tissues were embedded and cut into 3–5 μm-thick sections for hematoxylin-eosin (H&E) staining. Pathological scores of the lung tissues, including alveolar wall thickening, inflammatory cell infiltration, hemorrhage, edema, and necrosis, were evaluated as previously reported [19].

### 2.5. Full Length 16S rRNA Gene Sequencing and Bioinformatics Pipeline

Genomic DNA of BALF and fecal samples, negative control (control extraction with no sample) and mock community (Zymo, Tustin, CA, USA) were amplified using Kapa HiFi HotStart ReadyMix (Kapa Biosystems, Wilmington, MA, USA) with 16S rDNA primers: forward primer 27F (59-AGAGTTTGATCCTGGCTCAG-39) and reverse primer 1492R (59-TACGACTTAACCCCAATCGC-39). The PCR cycling protocol consisted of an initial denaturation at 95 °C for 1 min, followed by 20 cycles of 98 °C for 20 s, 55 °C for 12 s and 72 °C for 1 min, with a final extension at 72 °C for 5 min. The PCR products (*n* = 4 per group) were purified by 0.8× AMPure XP Beads (Beckman, Brea, CA, USA), quantified with a Qubit 4.0 fluorometer (Invitrogen, Carlsbad, CA, USA), and used to prepare DNA libraries with a Ligation Sequencing Kit SQK-LSK109 (Oxford Nanopore Technologies, Oxford, UK). The library was sequenced on a MinION Mk1B platform with an R9.4 flow cell (Oxford Nanopore Technologies, Oxford, UK). Raw reads were base-called, demultiplexed, and trimmed to remove adapters, primers, and primer links using Guppy base caller (v5.0.111). High-quality (min_score = 8) reads with lengths of between 1200 and 1800 bp were used for further analysis. The species composition distribution for each sample was estimated using Emu software [20], which uses Minimap (v2.24), the expectation maximization algorithm, and Emu v3.0 + database. Alpha diversity, beta diversity, clustering of samples, and visualization of species classification were performed using MicrobiomeAnalyst 2.0 as previously described [21]. KEGG pathway abundance of microbiota was predicted using PICRUST2 software [22].

### 2.6. Flow Cytometry and Enzyme-Linked Immunosorbent Assays

Fresh lung tissues were dispersed into single-cell suspensions by type 2 collagenase (1 mg/mL) and DNase I (100 μg/mL) at 37 °C for 1 h as previously described [23]. Single-cell suspensions were then centrifuged at 300× *g* for 10 min to pellet cells and remove erythrocytes by using BD Pharm Lyse lysing buffer (BD Biosciences, San Jose, CA, USA). Cells were blocked with TruStain αCD16/CD32 Fc-Block (BioLegend, San Diego, CA, USA), incubated at 22 °C for 10 min, and treated with antibiotics (1:50~1:100) in the dark for 15 min. Flow cytometry was performed on a BD FACSCanto II system (BD Biosciences, San Jose, CA, USA), and data were analyzed using FlowJo software 10.8.1 (BD Biosciences, San Jose, CA, USA). Gating strategies are provided in Figure S2. A list of antibodies used in this study is provided in Table S1. For IgA analysis, BALF secretory IgA (sIgA), BALF H1N1-specific IgA, and serum microbial reactivity IgA were measured using Mouse Secretory IgA, H1N1-specific IgA and Microbial reactivity IgA ELISA kits (MEIMIAN, Beijing, China) according to the manufacturers’ instructions. B cell activating factor (BAFF), A proliferation-inducing ligand (APRIL), and IL-21 levels of lung tissues were determined using Mouse BAFF, APRIL, and IL-21 ELISA kits (MEIMIAN, Beijing, China) according to the manufacturers’ instructions.

### 2.7. Statistical Analyses

Statistical analyses were performed using Prism 8.0 (GraphPad, Boston, MA, USA). The data on bodyweights, pathogen loads, and immune cell levels were presented as mean ± standard deviation. Log10-transformed values were used to express reductions in viral and bacterial loads for clarity and comparability. For multiple-group analysis, differences of bodyweights, pathogen loads, cells, Chao1, bacterial taxon abundances, antibodies, and cytokines were compared by one-way analysis of variance (ANOVA) using the Kruskal–Wallis test. For two-group analysis, data were compared by the Student’s *t*-test. The correlation analyses were performed using Spearman’s correlation analysis. A *p*-value of <0.05 was considered statistically significant.

## 3. Results

### 3.1. L. murinus Supplementation Reduces Susceptibility to Secondary MRSA Infection in IAV-Infected Mice

**Figure 1:**
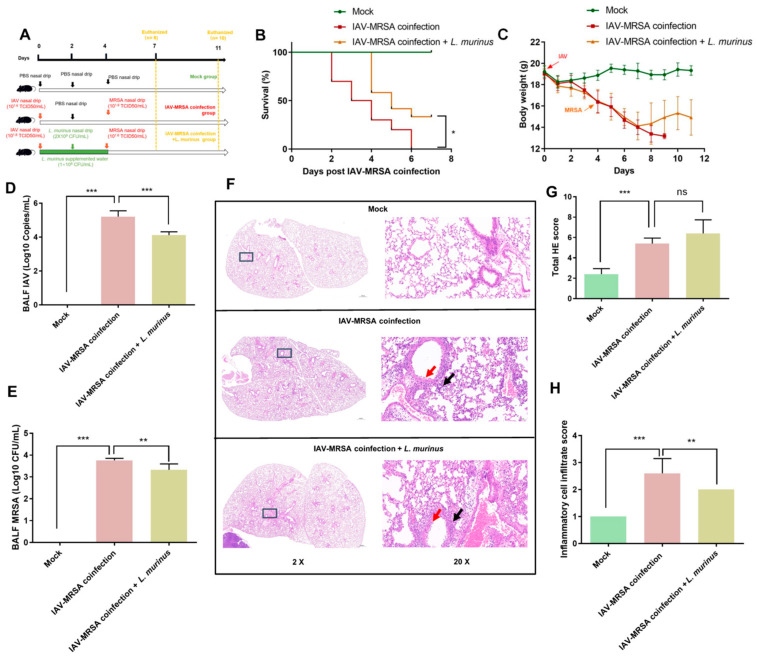
*L. murinus* supplementation reduces susceptibility to secondary MRSA infection in IAV-infected mice. (**A**) Establishment of *L. murinus* supplementation in secondary MRSA infection following IAV coinfection model. (**B**) The 7-day survival rate of IAV–MRSA coinfected mice (*n* = 10). (**C**) Bodyweight changes in mice during the experiment. (**D**) IAV load in BALF at 3 days post-coinfection (*n* = 5). (**E**) MRSA load in BALF at 3 days post-coinfection (*n* = 5). (**F**) H&E staining of the left lung at 3 days post-coinfection (*n* = 5). (**G**) Total H&E staining score of left lungs at 3 days post-coinfection (*n* = 5). (**H**) Inflammatory cell infiltrate score of left lungs at 3 days post-coinfection (*n* = 5). IAV, influenza A virus; MRSA, methicillin-resistant *Staphylococcus aureus*; HE staining, Hematoxylin and Eosin Staining. Bodyweight, IAV titer, and histologic scoring were compared by one-way analysis of variance (ANOVA), * *p* < 0.05, ** *p* < 0.01, *** *p* < 0.001, ns means no significant difference.

We performed a secondary MRSA infection after *L. murinus* supplementation in the respiratory tract and gut of IAV-infected mice. The survival rate at 7 days post-coinfection and lung injury at 3 days post-coinfection were measured to clarify the effect of the *L. murinus* on susceptibility to secondary MRSA infection, followed by IAV infection (Figure 1A). Data showed that the survival rate of the IAV–MRSA coinfection + *L. murinus* group was significantly increased compared with that of the IAV–MRSA coinfection group at 7 days post-coinfection (Figure 1B). The bodyweight of the IAV–MRSA coinfection + *L. murinus* group was increased compared to that of the IAV–MRSA coinfection group from 4 days post-coinfection. (Figure 1C). The IAV and MRSA load of BALF samples of IAV–MRSA coinfection + *L. murinus* group were significantly decreased compared with those of the IAV–MRSA coinfection group at 3 days post-coinfection (Figure 1D,E). H&E staining of lung tissues at 3 days post-coinfection showed lung injury features such as inflammatory cell infiltration (black arrows) and bronchial epithelial cell damage (red arrows) in both the IAV–MRSA coinfection group and IAV–MRSA coinfection + *L. murinus* group (Figure 1F). The total HE scores of lung tissues in the IAV–MRSA coinfection + *L. murinus* group showed no significant difference from those of the IAV–MRSA coinfection group (Figure 1G), but the inflammatory cell infiltration scores were significantly lower (Figure 1H).

### 3.2. L. murinus Supplementation Enhanced Lung IgA Response and the Levels of TI IgA Pathway Markers in IAV Infected Mice

**Figure 2:**
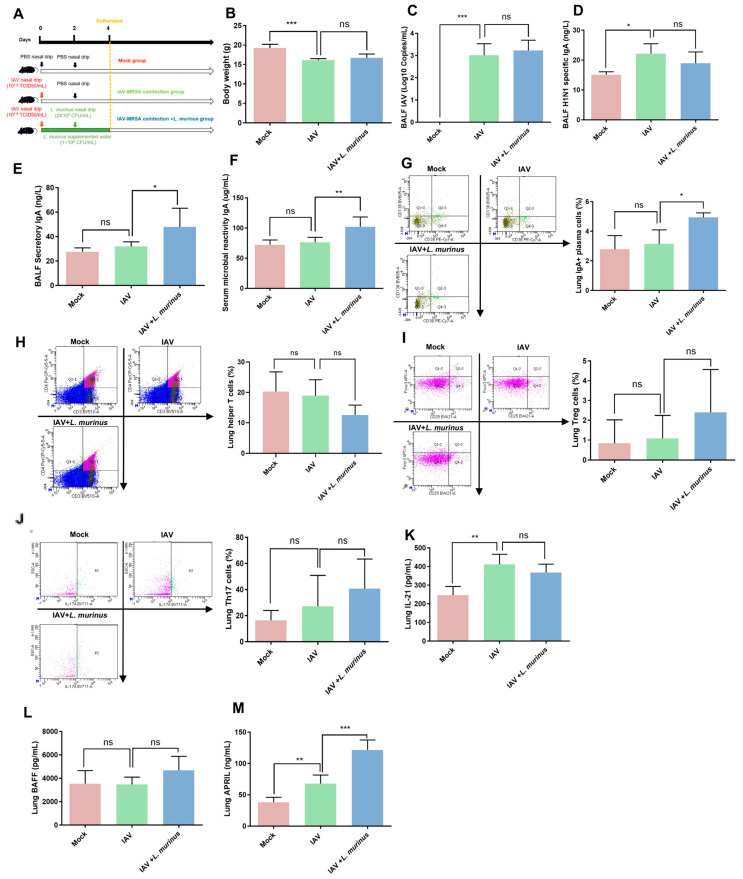
*L. murinus* supplementation enhanced the TI IgA response in the lungs of IAV-infected mice. (**A**) *L. murinus* supplementation in IAV-infected mice. (**B**) Bodyweight changes in mice at 4 days post-IAV infection. (**C**) IAV load in BALF at 4 days post-IAV infection. (**D**) H1N1-specific IgA levels in BALF at 4 days post-IAV infection. (**E**) Secretory IgA levels in BALF at 4 days post-IAV infection. (**F**) Microbial reactive IgA levels in serum at 4 days post-IAV infection. (**G**) IgA+ plasma cell levels in lungs at 4 days post. (**H**) Helper T cell levels in lungs at 4 days post-IAV infection. (**I**) Treg cell levels in the lung at 4 days post-IAV infection. (**J**) Th17 cell levels in the lung at 4 days post-IAV infection. (**K**) IL-21 levels in lungs at 4 days post-IAV infection. (**L**) BAFF levels in lungs at 4 days post-IAV infection. (**M**) APRIL levels in lungs at 4 days post-AV infection; IAV, influenza A virus; BAFF, B-cell activating factor of the TNF family; APRIL, A proliferation-inducing ligand. Bodyweight, IAV titer, cytokines, and cell levels were compared by one-way analysis of variance (ANOVA), *n* = 6 per group, * *p* < 0.05, ** *p* < 0.01, *** *p* < 0.001, ns means no significant difference.

The above results suggested that *L. murinus* supplementation after IAV infection can reduce susceptibility to secondary MRSA infection in IAV-infected mice, but the potential therapeutic targets remain unclear. To explore the related mechanisms, we investigated the bodyweight, IAV load, and lung IgA response in IAV-infected mice after *L. murinus* supplementation (Figure 2A). The results showed that *L. murinus* supplementation had no significant effect on bodyweight depression and IAV load elevation in IAV-infected mice (Figure 2B,C). For lung IgA response, the level of BALF H1N1-specific IgA in the IAV group was significantly higher than that in the Mock group, while there was no significant difference compared with the IAV + *L. murinus* group (Figure 2D). The levels of BALF secreted IgA, serum microbial reactive IgA and lung IgA + plasma cell in the IAV group were not significantly different compared with those in the Mock group, whereas they were significantly higher in the IAV + *L. murinus* group than in the IAV group (Figure 2E–G). To elucidate the mechanisms involved, we characterized the TD/TI IgA pathways in *L. murinus* supplemented mice. The data showed that the levels of factors related to the TD IgA pathway, including helper T cell, Treg, Th17 cell, and IL-21 levels, were not significantly different in the lungs of the IAV + *L. murinus* group compared to the IAV group (Figure 2H–K). For TI IgA pathways, data showed no significant difference in the level of lung BAFF between the three groups (Figure 2L), but the level of lung APRIL was significantly increased in the IAV + *L. murinus* group compared to the IAV group (Figure 2M).

### 3.3. L. murinus Supplementation Reversed Gut Microbiota Dysbiosis, but Had No Effect on LRT Microbiota Dysbiosis in IAV-Infected Mice

**Figure 3:**
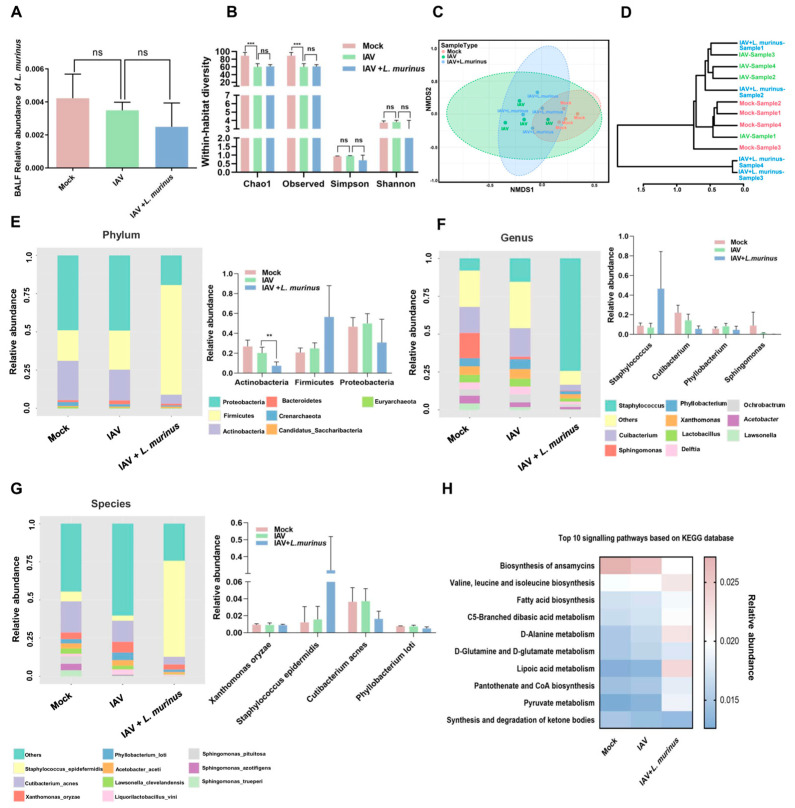
*L. murinus* supplementation showed no effect on IAV-induced LRT microbiota dysbiosis in mice. (**A**) The relative abundance of *L. murinus* in LRT microbiota at 4 days post-IAV infection. (**B**) Diversity analysis of LRT microbiota in mice at 4 days post-IAV infection. (**C**) NMDS analysis of LRT microbiota at 4 days post-IAV infection. (**D**) Cluster analysis of LRT microbiota in mice at 4 days post-IAV infection. (**E**) Top 10 bacteria at the phylum level of LRT microbiota in mice at 4 days post-IAV infection. (**F**) Top 10 bacteria at the genus level of LRT microbiota in mice at 4 days post-IAV infection. (**G**) Top 10 bacteria at the species level of LRT microbiota in mice at 4 days post-IAV infection. (**H**) Top 10 KEGG metabolism pathways prediction of LRT microbiota in mice at 4 days post-IAV infection. *n* = 4 per group. IAV, influenza A virus; LRT, lower respiratory tract. Diversity index and the relative abundance of bacteria were compared by one-way analysis of variance (ANOVA), ** *p* < 0.01, *** *p* < 0.001, ns means no significant difference.

Considering that the host microbiota is an important target for modulating the lung IgA response, we investigated the characteristics of the lower respiratory tract (LRT) and gut microbiota in IAV-infected mice with *L. murinus* supplementation. For LRT microbiota, the relative abundance of *L. murinus* in the IAV group showed a slight decrease compared to the mock group, but supplementation with *L. murinus* did not increase the relative abundance of *L. murinus* in the IAV + *L. murinus* group (Figure 3A). Moreover, IAV infection significantly reduced the richness (Chao1 and Observed index) and influenced the structure (NMDS analysis and cluster analysis) of LRT microbiota; however, these effects were not reversed by *L. murinus* supplementation (Figure 3B–D). We then analyzed the effect of *L. murinus* supplementation on the taxonomic profiles of the LRT in IAV-infected mice. At the phylum level, the core microbiome was dominated by *Proteobacteria*, *Firmicutes*, and *Actinobacteria*, with no significant differences between the three groups. (Figure 3E). At the genus and species levels, the core microbiome was dominated by *Staphylococcus*, *Cutibacterium*, *Phyllobacterium*, *Sphingomonas*, *Xanthomonas oryzae*, *Staphylococcus epidermidis*, *Cutibacterium acnes*, and *Phyllobacterium loti*, with no significant differences in their relative abundance between the three groups (Figure 3F,G). The metabolic function prediction of LRT microbiota showed that IAV infection decreased the level of biosynthesis of ansamycins and increased the level of D-Alanine metabolism compared with the Mock group, but these effects were not reversed by *L. murinus* supplementation (Figure 3H).

**Figure 4:**
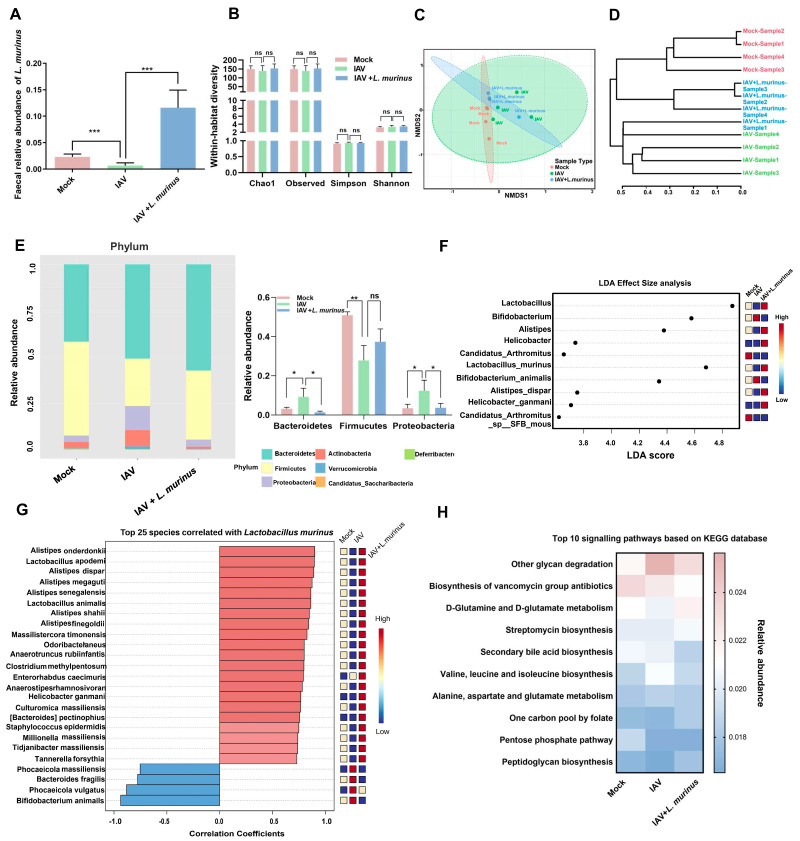
*L. murinus* supplementation repaired IAV-induced gut microbiota dysbiosis in mice. (**A**) The relative abundance of *L. murinus* in gut microbiota at 4 days post-IAV infection. (**B**) Diversity analysis of gut microbiota in mice at 4 days post-IAV infection. (**C**) NMDS analysis of gut microbiota at 4 days post-IAV infection. (**D**) Cluster analysis of gut microbiota in mice at 4 days post-IAV infection. (**E**) TOP 10 bacteria at the phylum level of gut microbiota at 4 days post-IAV infection. (**F**) The LEfSe analysis for the genus and species-level microbiome of the gut microbiota in mice 4 days after IAV infection. (**G**) Correlations between *L. murinus* and the top 25 gut bacterial taxa at the species level in the gut. (**H**) Top 10 KEGG metabolism pathways prediction of gut microbiota in mice at 4 days post-IAV infection. Diversity index and the relative abundance of bacteria were compared by one-way analysis of variance (ANOVA), *n* = 6 per group, * *p* < 0.05, ** *p* < 0.01, *** *p* < 0.001, ns means no significant difference. LDA effect size analysis was used to identify biomarkers by comparing abundance between groups (Wilcoxon test *p* < 0.01 and |log10(LDA)| > 3).

For gut microbiota, the relative abundance of *L. murinus* in the IAV group showed a significant decrease compared to the mock group, and supplementation with *L. murinus* greatly increased the relative abundance of *L. murinus* in IAV-infected mice (Figure 4A). However, there were no significant differences in the diversity and richness of gut microbiota among the three groups (Figure 4B). Cluster analysis and NMDS analysis results showed that IAV infection strongly influenced the gut microbiota, and this effect was reversed by *L. murinus* supplementation, as results of IAV + *L. murinus* samples clustered away from the IAV group and closed to the Mock group according to cluster analysis (Figure 4C,D). The effect of *L. murinus* supplementation on taxonomic profiles at different levels of classification was also investigated. At the phylum level, IAV infection reduced the relative abundance of *Firmicutes* but increased the relative abundance of *Proteobacteria* and *Bacteroidetes* compared with the Mock group, and this effect was reversed by *L. murinus* supplementation (Figure 4E). The LDA effect size analysis for multiple group comparisons showed that IAV infection reduced the relative abundance of *Lactobacillus* (*Lactobacillus murinus*) and *Alistipes* (*Alistipes dispar*), but increased that of *Bifidobacterium* (*Bifidobacterium animalis*) compared with the Mock group, and all the changes were reversed by *L. murinus* supplementation (Figure 4F). Further, we showed that the top 25 species are significantly associated with the relative abundance of *L. murinus.* We found that most of the species that are strongly positively correlated with *L. murinus*, such as *Alistipes onderdonkii*, *Lactobacillus apodemi*, *Alistipes dispar*, etc., were reduced in the IAV group compared to the Mock group and increased in the IAV + *L. murinus* group compared to the IAV group. In contrast, species strongly negatively correlated with *L. murinus*, such as *Phocaeicola massiliensis*, *Bacteroides fragilis*, *Phocaeicola vugatus*, and *Bifidobacterium animalis,* were increased in the IAV group compared to the Mock group and decreased in the IAV + *L. murinus* group compared to the IAV group (Figure 4G). The metabolic function prediction of gut microbiota showed that IAV infection decreased the levels of peptidoglycan biosynthesis, the pentose phosphate pathway, secondary bile acid biosynthesis, D-glutamine and D-glutamate metabolism, and biosynthesis of the vancomycin group antibiotic compared with the Mock group. In particular, the level of peptidoglycan biosynthesis and D-glutamine and D-glutamate metabolism were reversed by *L. murinus* supplementation (Figure 4H).

### 3.4. Gut Microbiota and Lung IgA Response Show Correlation in Mice

**Figure 5:**
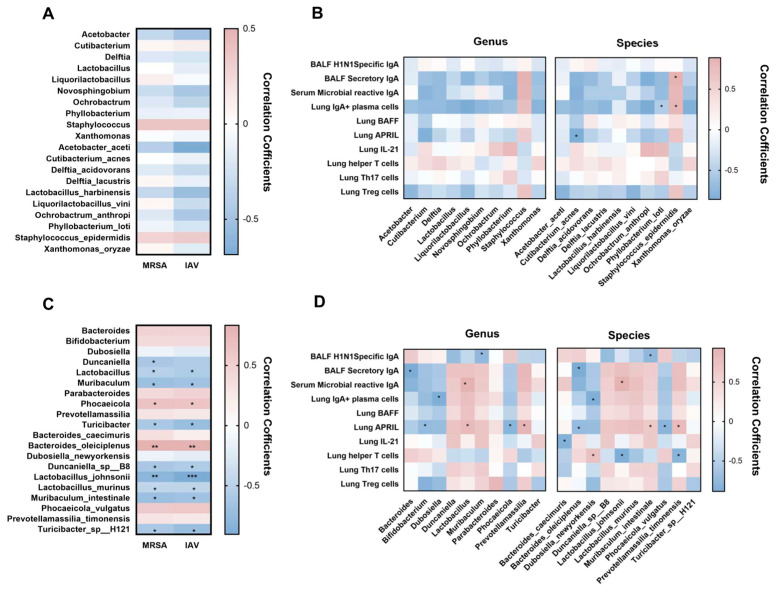
The gut microbiota was significantly correlated with pathogen load and IgA response in the lung. (**A**) Correlations between pathogen load and the top 10 LRT bacterial taxa at the genus and species level. (**B**) Correlations between lung IgA response and top 10 LRT bacterial taxa at the genus and species level. (**C**) Correlations between pathogen load and the top 10 gut bacterial taxa at the genus and species level. (**D**) Correlations between lung IgA response and top 10 gut bacterial taxa at the genus and species level. MRSA, methicillin-resistant *Staphylococcus aureus*; IAV, influenza A virus; LRT, lower respiratory tract. * *p* < 0.05, ** *p* < 0.01, *** *p* < 0.001. Blue indicates a negative correlation; red indicates a positive correlation.

To investigate the relationship between microbiota alterations induced by *L. murinus* supplementation and lung IgA response. Here, we used Spearman correlation analysis to explore the relationship between the relative abundance of the top 10 bacterial genera/species in LRT/gut and the levels of lung IgA response indicators and pathogen load. The results of correlation analysis between LRT microbiota and viral/bacterial load showed that there was no significant correlation between the top 10 bacterial genera/species and MRSA/IAV load (Figure 5A). Moreover, according to the results of correlation analysis between LRT microbiota and immune parameters, the BALF secretory IgA was significantly positively correlated with *Staphylococcus epidermidis.* The lung IgA+ plasma cell was significantly positively correlated with *Staphylococcus epidermidis,* but significantly negatively correlated with *Phyllobacterium loti*. The lung APRIL was significantly negatively correlated with *Cutiabacterium acnes* (Figure 5B).

The results of correlation analysis between gut microbiota and viral/bacterial load showed that the MRSA/IAV load was significantly positively correlated with *Phocaeicola* and *Bacteroides oleiciplenus*, but significantly negatively correlated with *Lactobacillus*, *Muribaculum*, *Turicibacter*, *Duncaniella* sp *B8*, *Lactobacillus johnsonii*, *Lactobacillus murinus, Muribaculum intestinale*, and *Turicibacter* sp *H121* (Figure 5C). Further correlation analysis between gut microbiota and immune parameters also showed that the BALF H1N1-specific IgA was significantly negatively correlated with *Muribaculum* and *Muribaculum intestinale*. The BALF secretory IgA was significantly negatively correlated with *Bacteroides* and *Bacteroides oleiciplenus*; the serum microbial reactive IgA was significantly positively correlated with *Lactobacillus* and *Lactobacillus johnsonii*. The lung IgA+ plasma cell was significantly negatively correlated with *Dubosiella* and *Dubosiella newyorkensis*. The lung APRIL was significantly negatively correlated with *Bifidobacterium*, *Phocaeicola*, *Bacteroides oleiciplenus* and *Phocaeicola vulgatus*, which were positively correlated with *Lactobacillus*, *Parabacteroides*, *Lactobacillus johnsonii*, and *Prevotella massilia timonensis*. The lung IL-21 was significantly negatively correlated with *Bacteroides caecimuris*. The lung T helper cell was significantly negatively correlated with *Lactobacillus johnsonii* and *Prevotellamassilia timonensis*, which was positively correlated with *Dubosiella newyorkensis* (Figure 5D).

### 3.5. TI-IgA Response in the Lung Is Dependent on Gut Microbiota

**Figure 6:**
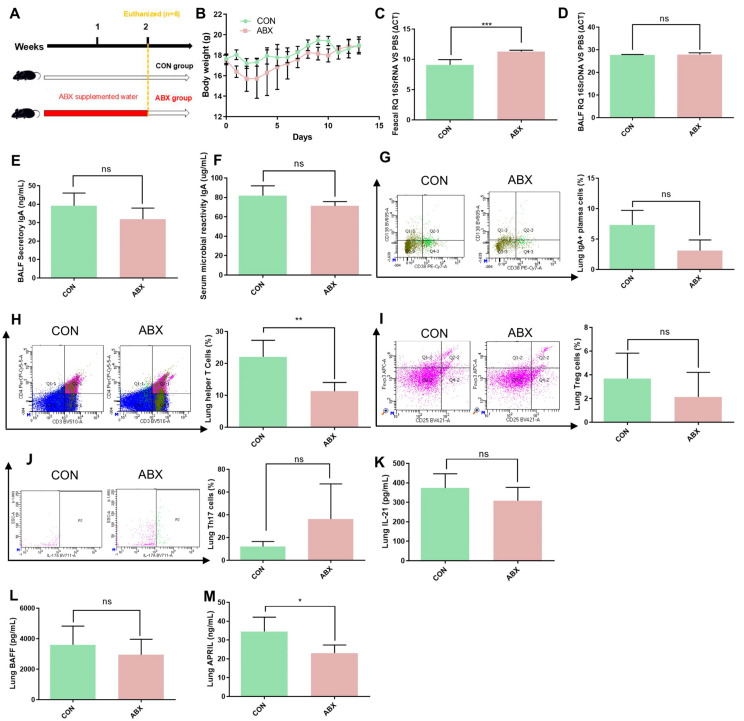
ABX treatment decreased the TI-IgA response in the lungs of mice. (**A**) ABX treatment in mice. (**B**) Bodyweight changes in mice during the experiment. (**C**) Intestinal bacterial load in mice. (**D**) Lower respiratory tract bacterial load in mice. (**E**) Secretory IgA levels in BALF in mice. (**F**) Microbial reactive IgA levels in serum in mice. (**G**) IgA+ plasma cell levels in the lungs of mice. (**H**) Helper T cell levels in the lungs of mice. (**I**) Treg cell levels in the lungs of mice. (**J**) Th17 cell levels in the lungs of mice. (**K**) IL-21 levels in the lungs of mice. (**L**) BAFF levels in the lungs of mice. (**M**) APRIL levels in the lungs of mice. IAV, influenza A virus; BAFF, B-cell activating factor of the TNF family; APRIL, A proliferation-inducing ligand. Bodyweight, IAV titer, cytokine, and cell levels were compared by Student’s *t* test, *n* = 6 per group, * *p* < 0.05, ** *p* < 0.01, *** *p* < 0.001, ns means no significant difference.

To further investigate the relationship between lung response and gut microbiota, we used a cocktail of antibiotics to deplete the gut microbiota of mice (Figure 6A). The bodyweight results showed a transient weight loss induced by antibiotic treatment on days 0–5, but then recovered (Figure 6B). Two weeks of antibiotic treatment significantly depleted the gut microbiota (Figure 6C), but had no significant effect on LRT microbiota (Figure 6D). Data showed the levels of BALF secretory IgA, serum microbial reactive IgA, and IgA+ plasma cells were slightly lower in the ABX group than in the CON group (Figure 6E–G). Moreover, we examined the levels of TD-IgA response indicators in lung and showed the level of lung helper T cells showed significant decrease in ABX group compared to CON group (Figure 6H), but there was no significant difference in lung helper T cells, Treg cells, Th17 cells and IL-21 levels between antibiotic treated mice and control mice (Figure 6I–K). We also quantified TI-IgA response indicators in lung tissue supernatants and showed that the level of BAFF showed no significant change (Figure 6L), but that of APRIL was significantly decreased in antibiotic-treated mice compared with samples from control mice (Figure 6M).

## 4. Discussion

Supplementation with *Lactobacillus* spp. is known to as a viable strategy for the treatment or prevention of infectious diseases through barrier enhancement, pathogen competition, immune modulation, and metabolic regulation [24]. Our previous study showed that the relative abundance of *L. murinus* was significantly reduced in the respiratory tract and gut of IAV-infected mice, and negatively correlated with the severity of IAV–MRSA coinfection pneumonia. However, the role and mechanism of *L. murinus* in the susceptibility to secondary MRSA pneumonia following IAV infection remained unclear. Here, we focused on the alterations of susceptibility to secondary MRSA pneumonia, host microbiota and lung TI/TD IgA response pathways by administration of *L. murinus* in IAV-infected mice, and investigated the relationship between lung IgA response and gut microbiota.

Seven-day mortality rates, histopathological evaluation, and pathogen load are important indicators for evaluating susceptibility to secondary bacterial infection in IAV-infected mice [25,26]. In this study, results showed that *L. murinus* supplementation significantly improved survival rates and alleviated lung inflammatory cell infiltration in IAV–MRSA coinfected mice. Interestingly, *L. murinus* supplementation reduced the pathogen load of both BALF IAV and MRSA in IAV–MRSA coinfected mice, but showed no significant effect on BALF IAV load in IAV-infected mice. These results suggested that the therapeutic target of *L. murinus* supplementation is MRSA rather than IAV. Similar phenomena have been observed in other studies, such as pre-treatment of *Cryptobacillus hidradii nematodes* with *Lactobacillus casei* Lb21, which reduced their susceptibility to MRSA [27]. The relevant mechanisms have yet to be explored.

Our previous studies found that the relative abundance of *L. murinus* was negatively correlated with MRSA loads and positively correlated with lung B cells in IAV–MRSA coinfected mice [6]. The activation and maturation of B cells generate the largest plasma cell population in the body, which secretes IgA [28]. These results suggested that the host IgA response to MRSA may be a key target for *L. murinus* supplementation to alleviate IAV–MRSA coinfection. As we expected, *L. murinus* supplementation increased total secretory IgA levels in the BALF, microbe-reactive IgA levels in blood, and IgA+ plasma cells in the lungs of IAV-infected mice in this study. IgA is mainly produced by IgA+ plasma cells through both TD and TI pathways and fights bacterial infections through inhibiting pathogen colonization, promoting colonization by commensal bacteria, and influencing bacterial metabolism [29]. Moreover, our results showed that *L. murinus* supplementation significantly increased APRIL levels in the lungs of IAV-infected mice. APRIL is a key member of the innate CD40 ligand-like factors and plays an important role in TI-IgA pathways, including B cell survival, proliferation, and Ig class switching [30]. On the contrary, *L. murinus* supplementation showed no significant effect on helper T cells, Treg cells, Th17 cells, and IL-21 levels in the lungs of IAV-infected mice in this study, which are important indicators for activation of TD IgA pathways. The above results demonstrated that *L. murinus* supplementation enhanced lung TI IgA response in IAV-infected mice.

Considering that the host microbiota plays an important role in the IgA response [31]. In this study, we investigated the effects of *L. murinus* supplementation on LRT and gut microbiota in IAV-infected mice to elucidate the related mechanisms. For LRT microbiota, data showed that IAV infection significantly reduced richness, influenced structure and altered composition of LRT microbiota, but *L. murinus* supplementation could not restore them. For gut microbiota, *L. murinus* supplementation restored IAV-induced gut microbiota dysbiosis. Notably, *Firmicutes/Bacteroidetes* (F/B) ratio was decreased, and the relative abundance of *Proteobacteria* was increased in the gut of IAV-infected mice, and this effect was reversed by *L. murinus* supplementation. Studies have shown that the increased or decreased prevalence of the F/B ratio and the increased prevalence of *Proteobacteria* have been considered as a potential diagnostic signature of dysbiosis and disease risk [32,33]. The LEfSe analysis data showed that the relative abundance of *Lactobacillus* and *Alistipes* increased in the gut of IAV-infected mice after *L. murinus* supplementation. Moreover, Spearman correlation analysis showed that *L. murinus* was strongly positively correlated with *Alistipes* species. Recent studies have demonstrated the beneficial effects of probiotic *Lactobacillus* on lung bacterial infection and identified *Alistipes* as potential probiotics to produce short-chain fatty acids and reduce the multidrug-resistant organism colonization [34,35]. Metabolic function prediction of gut microbiota showed that the reduction in peptidoglycan biosynthesis and D-glutamine and D-glutamate metabolism in IAV-infected mice was reversed by *L. murinus* supplementation. The synthesis of peptidoglycan, which, as multivalent antigens, could activate the TI-IgA response, requires glutamine uptake by the bacteria [36,37]. Spearman correlation analysis showed that the BALF secretory IgA was significantly negatively correlated with *Bacteroides* (*Bacteroides oleiciplenus*), and the serum microbial reactive IgA was significantly positively correlated with *Lactobacillus* (*Lactobacillus johnsonii*). *Bacteroides oleiciplenus* was identified as a unique potential pathogen and was increased in COVID-19 patients, while *Lactobacillus johnsonii*, as a probiotic, could increase average daily weight gain of calves and increase serum IgA levels [38,39]. This suggests that *L. murinus* supplementation can regulate the composition of the gut microbiota, which was strongly correlated with the lung IgA response.

To further investigate the relationship between IgA production in the lung and gut microbiota, we depleted the gut microbiota of mice with ABX treatment and detected lung IgA responses. The results showed that ABX treatment significantly reduced gut bacteria load and decreased IgA production in the lungs. Previous studies have similarly demonstrated a negative correlation between clinical antibiotic treatment and lung IgA levels and an increased susceptibility to lung *Pseudomonas aeruginosa* infection in ABX-treated mice [18]. We found that ABX treatment significantly reduced the APRIL level in the lungs. APRIL, as a TI IgA pathway factor, plays an important role in IgA class transformation and recombination via transmembrane activator and calmodulin cyclin ligand interaction factor and B cell maturation antigen. The above results suggest that the TI-IgA response in the lung is dependent on gut microbiota. In summary, *L. murinus* supplementation significantly reduced the susceptibility to MRSA in IAV-infected mice in this study. However, this is limited to the use of simultaneous intestinal and respiratory *L. murinus* supplementation. Future research needs to separately supplement *L. murinus* in the respiratory or intestinal tract to further explore the relevant antibacterial mechanisms. This study represents a preclinical mouse model investigation, and translation of these findings to human applications requires further extensive research. Additionally, the strain used in this study has not undergone a safety evaluation. In subsequent research, we will conduct safety assessments of this strain to exclude potential risks of infection, pro-inflammatory effects, and off-target effects.

## 5. Conclusions

In this study, we demonstrated that *L. murinus* supplementation significantly alleviated pneumonia caused by IAV–MRSA coinfection, restored IAV-induced gut microbiota and enhanced lung TI IgA response in mice. In addition, we demonstrated that lung TI-IgA response was dependent on gut microbiota using an antibiotic-treated mouse model. Together, our findings suggest that *L. murinus* may contribute to reducing MRSA susceptibility following IAV infection, possibly through TI-IgA immune modulation, which warrants further investigation. Further studies should be conducted to independently evaluate the efficacy of *L. murinus* administered through the respiratory and intestinal tracts.

## Data Availability

The original contributions presented in this study are included in the article/Supplementary Materials. Further inquiries can be directed to the corresponding author.

## Supplementary Materials

The following supporting information can be downloaded at: https://www.mdpi.com/article/10.3390/microorganisms13071709/s1, Figure S1: Establishment of secondary MRSA pneumonia following IAV infection model in mice; Figure S2: Gating strategy for identification of Th17, Treg, helper T cell and IgA+ plasma cell by flowcytometry; Table S1: List of antibodies used in this study.

## Abbreviations

The following abbreviations are used in this manuscript:

MRSA	Methicillin-resistant *Staphylococcus aureus*
IAV	Influenza A virus
BALF	Bronchoalveolar lavage fluid
SIgA	Secretory IgA
TD	T cell-dependent
TI	T cell-independent
BAFF	B-cell activating factor
APRIL	A proliferation-inducing ligand
PR8	Influenza virus strain A/Puerto Rico/8/34 (H1N1)
TCID50	50% tissue culture infective dose
CFU	Colony forming units
SPF	Specific pathogen free
ABX	Antibiotic cocktails
H&E	Hematoxylin-eosin
