# Supplementary material for: Lactobacillus murinus Reduces Susceptibility to Secondary MRSA Infection in IAV-Infected Mice Through Promoting a T Cell-Independent IgA Response
Source: Microorganisms. 2025 Jul 21;13(7):1709. doi: 10.3390/microorganisms13071709 (PMC12299313; doi:10.3390/microorganisms13071709)
Supplement: Supplementary file 1 [file microorganisms-13-01709-s001.zip › microorganisms-3724087-supplementary-change Fig S1Fig S2 to Figure S1Figure S1.pdf]

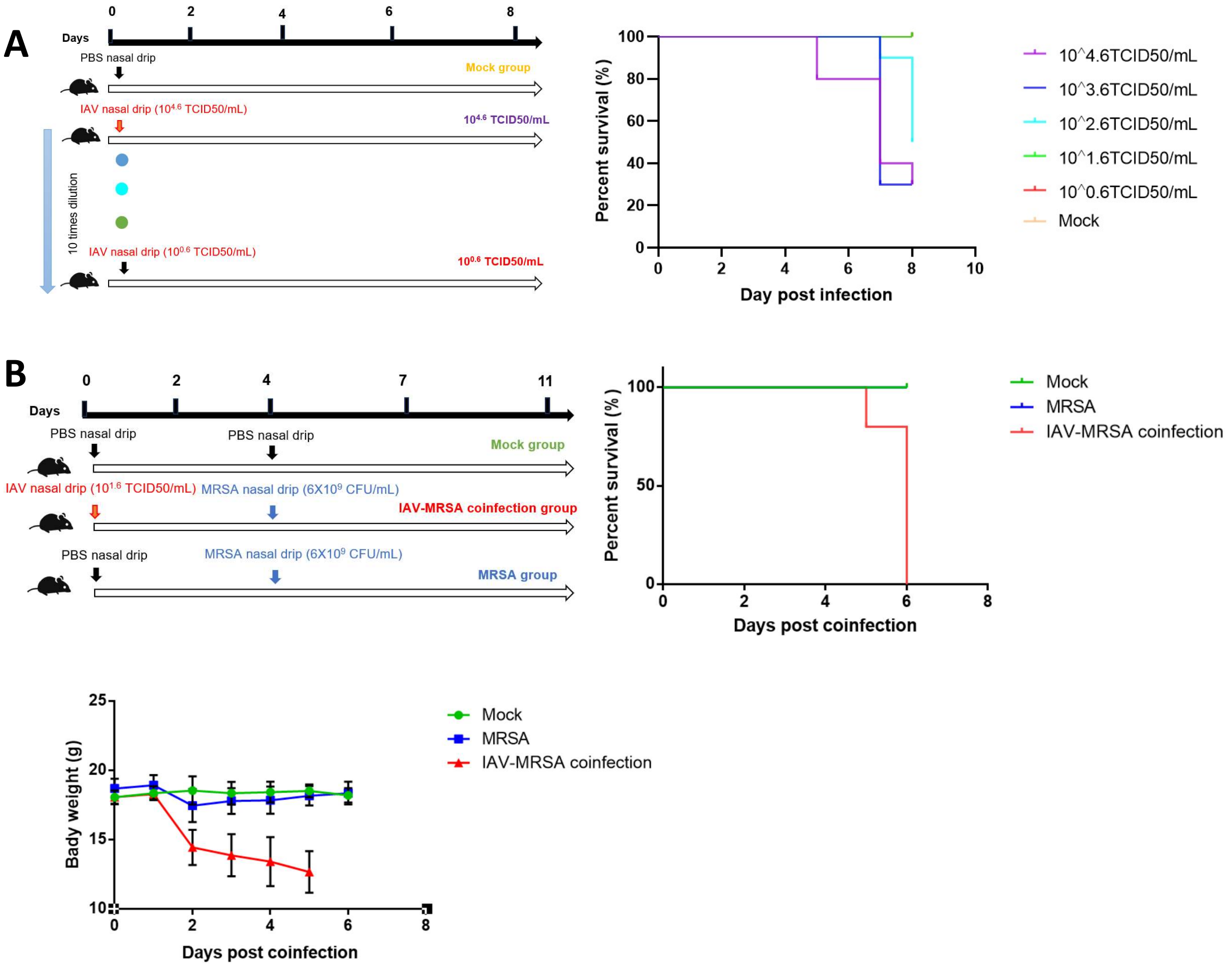

Figure S1 Establishment of secondary MRSA pneumonia following IAV infection model in mice. (A) Screening for sub-lethal doses of IAV in mice. (B) The 7-days survival rate and weight changes of secondary MRSA pneumonia following IAV infection model.

Gating strategies

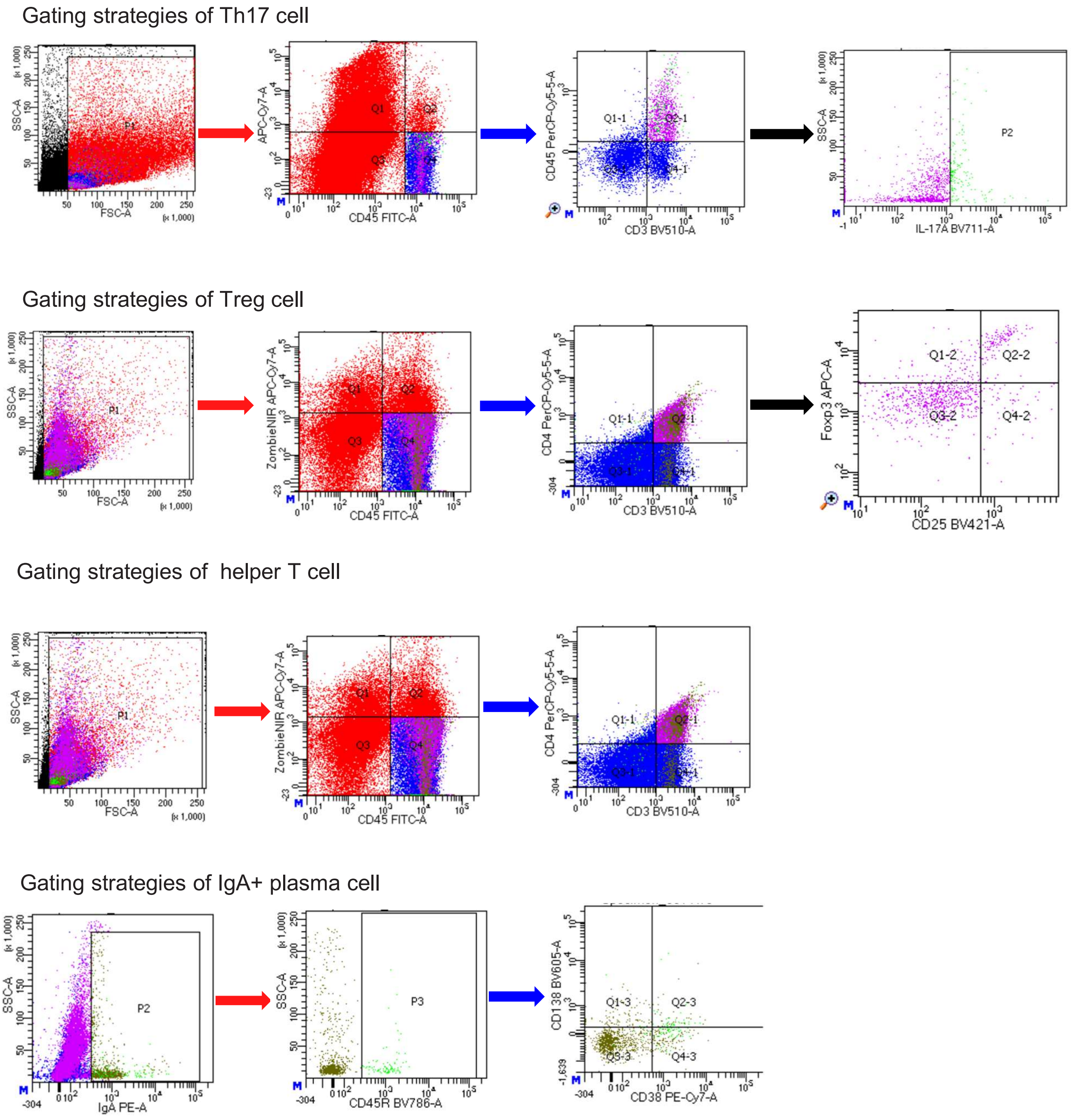

Figure S2 Gating strategy for identification of Th17, Treg, helper T cell and IgA+ plasma cell by flowcytometry.

Table S1: List of antibodies used in this study

ANTIBODIES	SOURCE	CATALOG #
FITC anti-mouse CD45 Antibody	Biolegend	157213
PerCP/Cyanine5.5 anti-mouse CD4 Antibody	Biolegend	100433
FITC anti-mouse CD45 Antibody	Biolegend	157213
Brilliant Violet 510™ anti-mouse CD3 Antibody	Biolegend	100233
Brilliant Violet 421™ anti-mouse CD25 Antibody	Biolegend	113705
Anti-Mouse/Rat Foxp3 Staining Set APC	eBioscience	77-5775-40
IgA Monoclonal Antibody (mA-6E1), PE,	eBioscience	12-4204-82
BD Horizon™ BV786 Rat Anti-Mouse CD45R/B220	BD Biosciences	563894
PE/Cyanine7 anti-mouse CD38 Antibody	Biolegend	102717
Brilliant Violet 605™ anti-mouse CD138 (Syndecan-1) Antibody	Biolegend	142515
PerCP/Cyanine5.5 anti-mouse CD45 Antibody	Biolegend	103131
Brilliant Violet 711™ anti-mouse IL-17A Antibody	Biolegend	506941
